# Global analysis of miRNA-mRNA regulation pair in bladder cancer

**DOI:** 10.1186/s12957-022-02538-w

**Published:** 2022-03-03

**Authors:** Xingchen Fan, Xuan Zou, Cheng Liu, Shuang Peng, Shiyu Zhang, Xin Zhou, Tongshan Wang, Wei Zhu

**Affiliations:** 1 Department of Oncology, First Affiliated Hospital of Nanjing Medical University, 300 Guangzhou Road, Nanjing, 210029 People’s Republic of China; 2 First Clinical College of Nanjing Medical University, 140 Hanzhong Road, Nanjing, 210029 People’s Republic of China; 3 Department of Gastroenterology, First Affiliated Hospital of Nanjing Medical University, 300 Guangzhou Road, Nanjing, 210029 People’s Republic of China

**Keywords:** miRNA, miRNA-mRNA regulation pairs, Bladder cancer

## Abstract

**Purpose:**

MicroRNA (miRNA) is a class of short non-coding RNA molecules that functions in RNA silencing and post-transcriptional regulation of gene expression. This study aims to identify critical miRNA-mRNA regulation pairs contributing to bladder cancer (BLCA) pathogenesis.

**Patients and methods:**

MiRNA and mRNA microarray and RNA-sequencing datasets were downloaded from gene expression omnibus (GEO) and the cancer genome atlas (TCGA) databases. The tool of GEO2R and R packages were used to screen differential miRNAs (DE-miRNAs) and mRNAs (DE-mRNAs) and DAVID, DIANA, and Hiplot tools were used to perform gene enrichment analysis. The miRNA-mRNA regulation pair were screened from the experimentally validated miRNA-target interactions databases (miRTarbase and TarBase). Twenty-eight pairs of BLCA tissues were used to further verify the screened DE-miRNAs and DE-mRNAs by quantitative reverse transcription and polymerase chain reaction (qRT-PCR). The diagnostic value of the miRNA-mRNA regulation pairs was evaluated by receiver operating characteristic curve (ROC) and decision curve analysis (DCA). The correlation analysis between the selected miRNA-mRNAs regulation pair and clinical, survival and tumor-related phenotypes was performed in this study.

**Results:**

After miRTarBase, the analysis of 2 miRNA datasets, 6 mRNA datasets, and TCGA-BLCA dataset, a total of 13 miRNAs (5 downregulated and 8 upregulated in BLCA tissues) and 181 mRNAs (72 upregulated and 109 downregulated in BLCA tissues) were screened out. The pairs of miR-17-5p (upregulated in BLCA tissues) and TGFBR2 (downregulated in BLCA tissues) were verified in the external validation cohort (28 BLCA vs. 28 NC) using qRT-PCR. Areas under the ROC curve of the miRNA-mRNA regulation pair panel were 0.929 (95% CI: 0.885–0.972, *p* < 0.0001) in TCGA-BLCA and 0.767 (95% CI: 0.643–0.891, *p* = 0.001) in the external validation. The DCA also showed that the miRNA-mRNA regulation pairs had an excellent diagnostic performance distinguishing BLCA from normal controls. Correlation analysis showed that miR-17-5p and TGFBR2 correlated with tumor immunity.

**Conclusions:**

The research identified potential miRNA-mRNA regulation pairs, providing a new idea for exploring the genesis and development of BLCA.

**Supplementary Information:**

The online version contains supplementary material available at 10.1186/s12957-022-02538-w.

## Introduction

Bladder cancer (BLCA) is among the most prevalent cancers worldwide, with 549,393 new cases reported in 2018 [1]. The risk of bladder cancer is approximately 1.1% for men and 0.27% for women [2]. BLCA can be divided into two major groups based on tumor stage: non-muscle-invasive bladder cancer (NMIBC) and muscle-invasive bladder cancer (MIBC) [3–5]. 20–30% of patients with NMIBC will progress to MIBC, and once the progression is identified, the patient's prognosis decreases [6–8]. Therefore, it is necessary to study the pathogenesis of bladder cancer.

MicroRNA (miRNA) is a class of short non-coding RNA molecules with 19 to 25 nucleotides in length, that functions in RNA silencing and post-transcriptional regulation of gene expression [9]. As a result, these mRNA molecules are silenced through the following processes: cleavage of the mRNA strand into two pieces, destabilization of the mRNA through shortening of its poly(A) tail, and less efficient translation of the mRNA into proteins by ribosomes [10, 11]. More and more studies focus on the regulatory pair of miRNA-mRNA, exploring its mechanism in the occurrence and development of diseases [12, 13].

We downloaded 2 miRNA datasets and 6 mRNA datasets from the Gene Expression Omnibus (GEO) database and combined the data from the TCGA database to screen for differential miRNAs (DE-miRNAs) and mRNA (DE-mRNAs) between BLCA and normal tissues. Interactions between DE-miRNAs and DE-mRNAs were determined using Tarbase and miRTarbase databases, where the miRNA-mRNA regulation pairs were validated experimentally. Then, we further validated the DE-miRNAs and DE-mRNAs in 28 pairs of BLCA tissues by qRT-PCR. The correlation analysis between the selected miRNA-mRNAs regulation pair and clinical, survival and tumor-related phenotypes was performed in this study. In summary, the interaction of the miRNA-mRNA regulatory pair had been researched in detail to provide a new idea or strategy for BLCA.

## Materials and methods

### Data acquisition and processing of miRNA and mRNA expression profiles

**Fig. 1:**
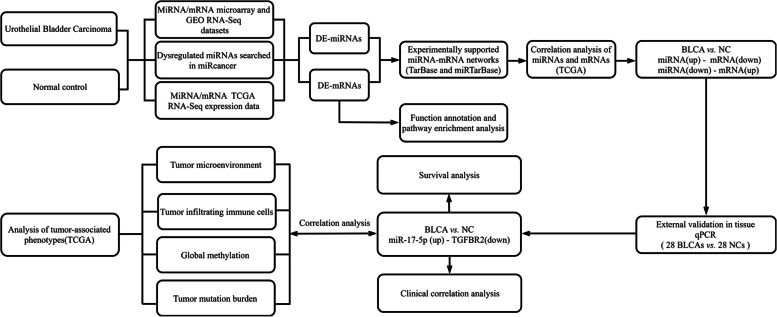
Flow chart for identifying the miRNA-mRNA regulatory pairs and the comprehensive analysis of regulatory pairs’ role in bladder cancer (BLCA)

We downloaded the miRNA and mRNA microarray expression datasets of BLCA from the Gene Expression Omnibus (GEO) database (http://www.ncbi.nlm.nih.gov/geo/). The TCGA-BLCA miRNA and mRNA sequencing expression profile and related clinicopathological data were downloaded from the GDC data portal of the National Cancer Institute (https://portal.gdc.cancer.gov/). An overview of the workflow steps is shown in Fig. 1. DE-miRNAs and DE-mRNAs were screened by the web analysis tool GEO2R in the GEO database (http://www.ncbi.nlm.nih.gov/geo/geo2r/) and “limma” and “edgeR” R packages.

### Identification and function analysis of miRNA-mRNA regulation pairs

TarBase is a reference database specifically designed to index experimentally supported miRNA targets, integrating information on cell type–specific miRNA gene regulation, while hundreds of thousands of miRNA-binding locations have been reported [14]. miRTarBase is a comprehensively annotated and experimentally validated database of miRNA-target interactions. Tarbase and miRTarBase databases were used to construct the miRNA-mRNA regulatory pairs [15]. Pearson correlation analysis of miRNA and mRNA in TCGA-BLCA was performed to filter the miRNA-mRNA regulation pairs. The online tool DAVID (http://david.abcc.ncifcrf.gov/) is a comprehensive tool for researchers and scholars to understand the biological significance behind multiple genes. The DIANA-MirPath is a miRNA pathway analysis web-server, and Hiplot is a comprehensive web platform for scientific data visualization [16]. The DAVID, DiANA-MirPath, and Hiplot were used for Gene Ontology (GO) functional analysis and Kyoto Encyclopedia of Genes Genomes (KEGG) pathways analysis.

### Ethical approval and Information of participants

The study was conducted in accordance with the guidelines of the Hospital Ethics Committee and approved by the Institutional Review Boards of the First Affiliated Hospital of Nanjing Medical University (ID: 2016-SRFA-148). Each participant signed informed consent in advance. All the participants of the experiment were recruited from the First Affiliated Hospital of Nanjing Medical University from 2016 to 2017. All samples were obtained from radical cystectomy and did not receive neoadjuvant chemotherapy before surgery. Then, formalin fixation and paraffin embedding (FFPE) specimens of tumor and normal tissues were obtained after operation and their pathological data were recorded. The clinical characteristics of the 28 BLCA patients are shown in Table 1.

**Table 1:** Clinicopathological and molecular features of BLCA patients

Variables	Number of cases	Rate (%)
	(***n*** = 28)	
**Age (years)**		
≤ 68	13	46.4
> 68	15	53.6
**Gender**		
Female	3	10.7
Male	25	89.3
**Muscle invasion**		
Present	19	67.9
Absent	9	32.1
**TNM stage**		
I–II	18	64.3
III–IV	10	35.7
**Lymph node metastasis**		
No	21	75
Yes	7	25

### Extraction of RNA and quantitative reverse transcription polymerase chain reaction

The TIANGEN RNAprep Pure FFPE kit (Tiangen, Beijing, China) was used to isolate total RNA from FFPE samples according to the manufacturer’s protocol. The acquired RNA from each sample was lysed in 100 μl RNase-free water and stored at – 80 °C until use. The concentration and purity of RNA samples were measured using the NanoDrop ND-1000 spectrophotometer (NanoDrop, Wilmington, DE, USA). External validation was performed by qRT-PCR using PrimeScript RT Reagent Kit (Takara) and SYBR Premix Ex Taq II (Takara) after adding a poly(A) tail to RNA by Poly(A) Polymerase Kit (Takara). The sequences of PCR primers are listed in Table S1. The expression levels of miRNAs and mRNAs in tissue samples were calculated using the 2^–ΔΔCt^ method (*RNU6B*[U6] as endogenous reference miRNA and GAPDH as endogenous reference mRNA for sample normalization; ΔCt = Ct miRNA − Ct normalizer; Ct: the threshold cycle) [17].

### Evaluation of interactions of miRNA-mRNA regulation pairs and tumor-relative phenotypes

Single sample gene set enrichment analysis (ssGSEA) is an extension of the GSEA method, which allows the definition of an enrichment score representing the absolute degree of enrichment of the gene set in each sample within a given dataset [18]. The data of ssGSEA was downloaded from UCSC Xena (https://xena.ucsc.edu/) to analyze the possible enrichment pathways of DE-miRNAs and DE-mRNAs. CIBERSORT is a general computational method for accurate estimation of immune components in tumor biopsy by combining support vector regression with prior knowledge of expression profile of purified leukocyte subsets [19]. We downloaded the infiltrating immune cell types data from the TCGA website and calculated using CIBERSORT (https://cibersort.stanford.edu/index.php/). The stromal and immune levels of TCGA-BLCA specimens were assessed using ESTIMATE software that uses gene expression characteristics to infer the proportion of stromal and immune cells in tumor specimens [20]. Tumor mutational burden (TMB) is a potential biomarker associated with therapeutic response to immune checkpoint inhibitors [21]. We downloaded TMB and DNA methylation profile data in TCGA-BLCA samples from the UCSC Xena platform (https://xena.ucsc.edu/). DNA methylation spectrum was measured using the Illumina Infinium Human Methylation450 platform.

### Statistical analysis

We used GraphPad Prism software v8.0, IBM SPSS Statistics v26.0 software (IBM Corporation, Armonk, NY, USA), and R language v3.6.3 (https://cran.r-project.org/) to analyze the data. The statistical criteria for screening DE-miRNAs and DE-mRNAs is |log2FC| > 0.58 and *p* < 0.05. The area under the ROC curve (AUC) and decision curve analysis (DCA) based on logistic regression were used to evaluate the diagnostic efficacy of miRNA-mRNA regulation pairs. The Pearson correlation method was used to calculate the correlation between DE-mRNAs or DE-miRNAs and tumor-related phenotypes. TCGA-BLCA prognostic data were grouped according to median survival time, and Kaplan-Meier survival curve analysis was performed.

## Results

### Identification of differentially expressed miRNAs and mRNAs in BLCA

**Fig. 2:**
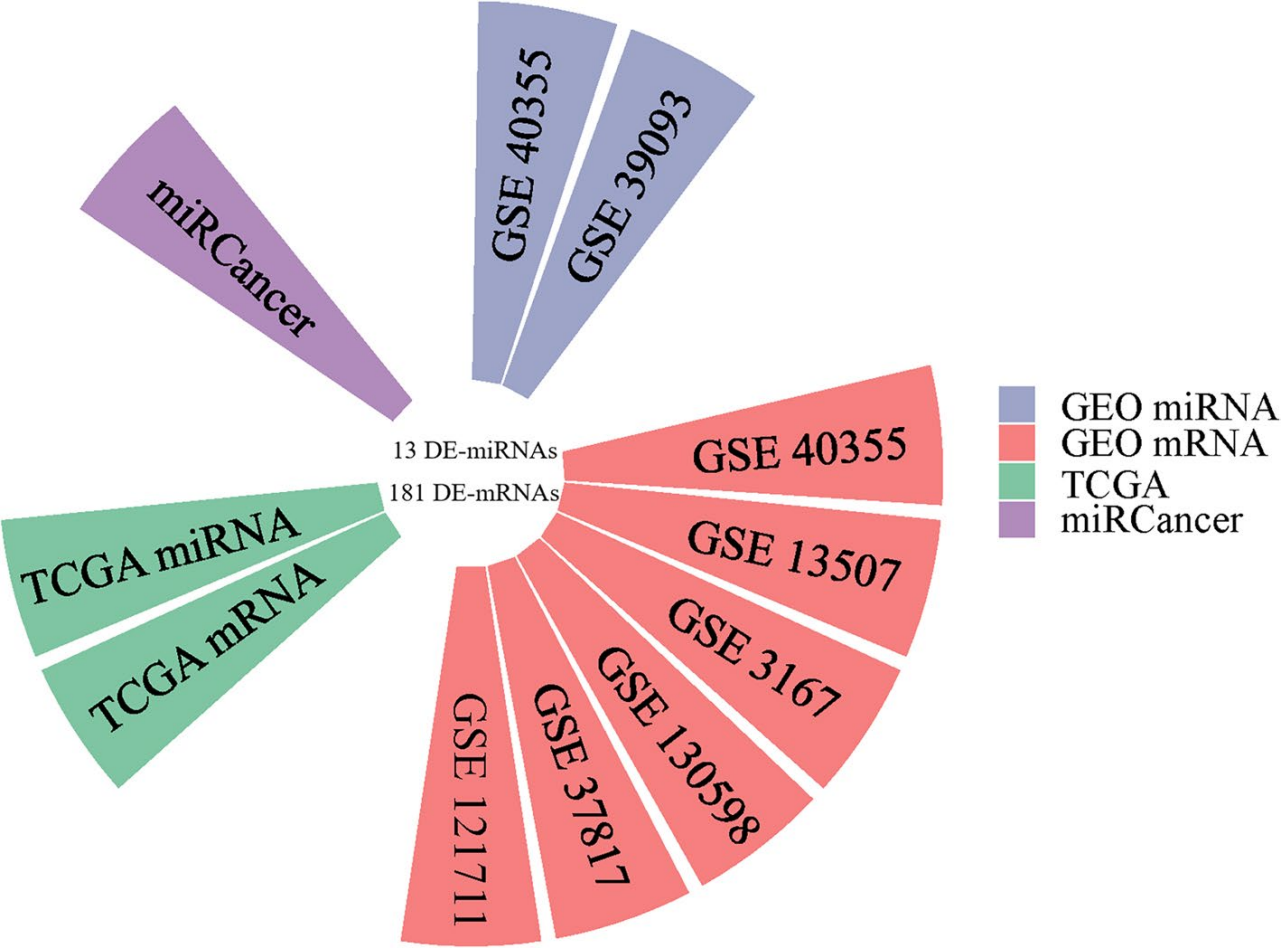
The circular bar chart showing the datasets from different sources for screening differentially expressed miRNAs and mRNAs. A total of 8 datasets from the GEO database were included in the study, including 6 mRNA expression microarray datasets and 2 microarray datasets. Combined with GEO, miRCancer, and TCGA databases, 13 miRNAs and 181 mRNAs were selected as DE-miRNAs and DE-mRNAs

We downloaded two miRNA and six mRNA expression datasets from the GEO database, and the information is shown in Table 2. As shown in Fig. 2, miRNAs and mRNAs with differences in each GEO dataset and TCGA dataset were selected as DE-miRNAs or DE-mRNAs. A total of 13 miRNAs (5 downregulated and 8 upregulated in BLCA tissues) and 181 mRNAs (72 upregulated and 109 downregulated in BLCA tissues) were selected as DE-miRNAs and DE-mRNAs (Table S2). KEGG pathway enrichment analysis revealed that the DE-miRNAs and DE-mRNAs enriched in the Pathways in cancer, proteoglycans in cancer, cGMP-PKG signaling pathway, etc. (Figure S1). The GO terms of the DE-miRNAs or DE-mRNAs were enriched in the cytosol, cellular component, protein phosphorylation, protein autophosphorylation, etc. These pathways are closely related to the occurrence and development of tumors (Figure S1).

**Table 2:** Information pertaining to the selected GEO datasets for BLCA

	Experiment type	Source name	GEO accession	Platform	Group	
					Tumor	Control
**microRNA expression**	Array	Tissue	GSE40355	GPL8227	16	8
			GSE39093	GPL8786	10	10
**mRNA expression**	Array	Tissue	GSE40355	GPL13497	16	8
			GSE13507	GPL6102	165	9
			GSE3167	GPL96	41	9
			GSE130598	GPL26612	24	24
			GSE37817	GPL6102	18	5
			GSE121711	GPL17586	8	10

### Screening of miRNA-RNA regulatory pairs associated with BLCA

**Fig. 3:**
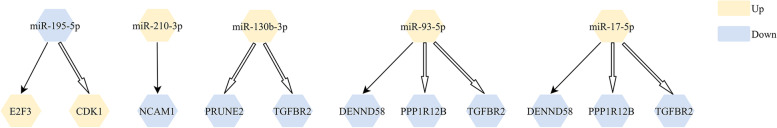
The screened miRNA-mRNA regulation pairs. A total of 11 miRNA-mRNA regulation pairs (miR-195-5p(down)/CDK1(up), miR-195-5p(down)/E2F3(up), miR-210-3p(up)/NCAM1(down), miR-93-5p(up)/DENND5B(down), miR-93-5p(up)/PPP1R12B(down), miR-93-5p(up)/TGFBR2(down), miR-130b-3p(up)/PRUNE2(down), miR-130b-3p(up)/TGFBR2(down), miR-17-5p(up)/DENND5B(down), miR-17-5p(up)/PPP1R12B(down), miR-17-5p(up)/TGFBR2(down)) were identified after verified in miRtarbase and Tarbase databases and the expression levels of 7 pairs of miRNA-mRNA (miR-195-5p(down)/CDK1(up), miR-130b-3p(up)/PRUNE2(down), miR-130b-3p(up)/TGFBR2(down), miR-93-5p(up)/PPP1R12B(down), miR-93-5p(up)/TGFBR2(down), miR-17-5p(up)/PPP1R12B(down), miR-17-5p(up)/TGFBR2(down)) in TCGA-BLCA showed significant negative correlation in Pearson’s correlation analysis (*p* < 0.05)

DE-miRNAs and DE-mRNAs were verified in miRtarbase and Tarbase databases, and 11 miRNA-mRNA regulation pairs (miR-195-5p(down)/CDK1(up), miR-195-5p(down)/E2F3(up), miR-210-3p(up)/NCAM1(down), miR-93-5p(up)/DENND5B(down), miR-93-5p(up)/PPP1R12B(down), miR-93-5p(up)/TGFBR2(down), miR-130b-3p(up)/PRUNE2(down), miR-130b-3p(up)/TGFBR2(down), miR-17-5p(up)/DENND5B(down), miR-17-5p(up)/PPP1R12B(down), miR-17-5p(up)/TGFBR2(down)) were identified (Fig. 3). The 11 pairs of miRNA-RNA were experimentally verified, and the expression levels of 7 pairs of miRNA-mRNA (miR-195-5p(down)/CDK1(up), miR-130b-3p(up)/PRUNE2(down), miR-130b-3p(up)/TGFBR2(down), miR-93-5p(up)/PPP1R12B(down), miR-93-5p(up)/TGFBR2(down), miR-17-5p(up)/PPP1R12B(down), and miR-17-5p(up)/TGFBR2(down)) in TCGA-BLCA showed a significant negative correlation in Pearson’s correlation analysis (*p* < 0.05) (Table S3).

### Validation of the expression of miRNAs and mRNAs in BLCA tissue

**Fig. 4:**
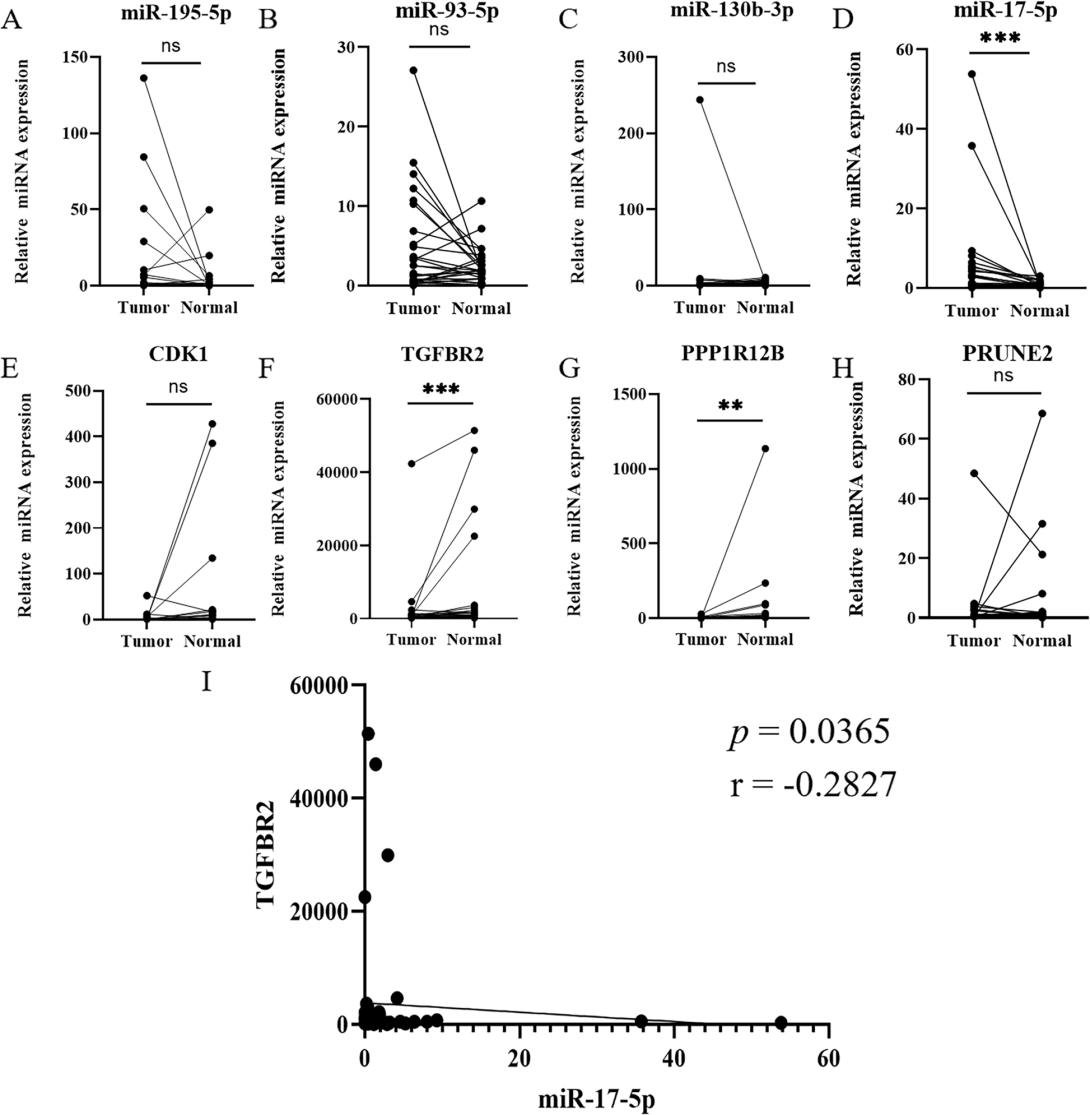
Validating the expression of the DE-miRNAs and DE-mRNAs by qRT-PCR (data are presented as mean ± SEM; *p < 0.05, **p < 0.01, ***p < 0.001). **A**: miR-195-5p; **B**: miR-93-5p; **C**: miR-130b-3p; **D**: miR-17-5p; **E**: CDK1; **F**: TGFBR2; **G**: PPP1R12B; **H**: PRUNE2; **I**: Pearson’s correlation analysis of miR-17-5p and TGFBR2

We further validated the 7 miRNA-mRNA regulation pairs in 28 pairs of matched tumors and adjacent normal tissues by qRT-PCR to validate the DE-miRNAs and DE-mRNAs. The expression of the TGFBR2(*p* = 0.001), PPP1R12B(*p* = 0.010) were downregulated in tumor tissues, while the expressions of miR-17-5(*p* < 0.000) were upregulated in tumor tissues (Fig. 4). At the same time, there was no significant difference between miR-195-5 (*p* = 0.068), miR-93-5 (*p* = 0.151), miR-130b-3 (*p* = 0.158), CDK1 (*p* = 0.084), and PRUNE2 (*p* = 0.733) expression in tumor tissues compared with normal tissues. We conducted a subgroup analysis on whether the expression of mRNA or miRNA was influenced by the presence of muscle invasion or lymph node invasion. As shown in Table S4, the results showed that the expression of mRNA or miRNA was not affected by the presence of muscle invasion or lymph node invasion. Based on Spearman’s correlation analysis of the pairs, miR-17-5p was significantly correlated with TGFBR2 expression (*p* = 0.0365, *r* = – 0.2827).

### Evaluation of the diagnostic value of miRNA-mRNA regulation pairs and the analysis of clinical and survival analysis in BLCA

**Fig. 5:**
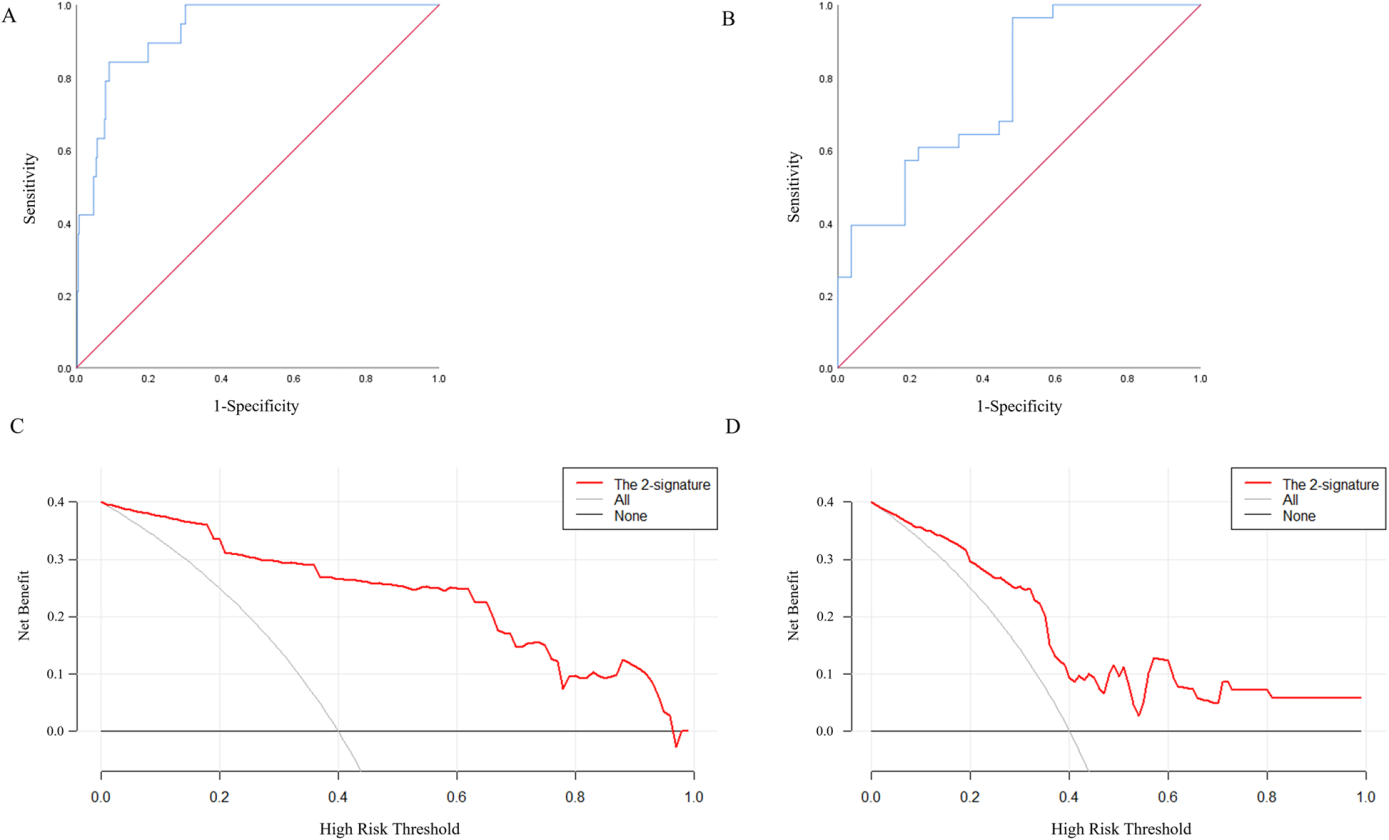
The ROC and DCA of the panel of miR-17-5p and TGFBR2 for discriminating BLCA patients from NCs. **A**: The ROC of the TCGA-BLCA (AUC = 0.929, 95% CI: 0.885-0.972, *p* < 0.0001); **B**: The ROC of the external validation (AUC = 0.767, 95% CI: 0.643–0.891, *p* = 0.001); **C**: The DCA of the TCGA-BLCA; **D**: The DCA of the external validation

MiR-17-5p and TGFBR2 were combined as a panel using the logistic regression analysis. As demonstrated in Fig. 5A, B, the AUC of the panel was 0.929 (95% CI: 0.885–0.972, *p* < 0.0001) in TCGA-BLCA and 0.767 (95% CI: 0.643–0.891, *p* = 0.001) in the external validation. The DCA showed that the miRNA-mRNA regulation pairs had a good diagnostic performance in distinguishing BLCA from normal patients (Fig. 5C, D). The expression of miR-17-5p and TGFBR2 showed no significant difference in age, gender, and TNM stage, and there was no significant correlation with prognosis (Figure S2).

### Analysis of tumor-related phenotypes associated with miRNA-mRNA regulation pairs

**Fig. 6:**
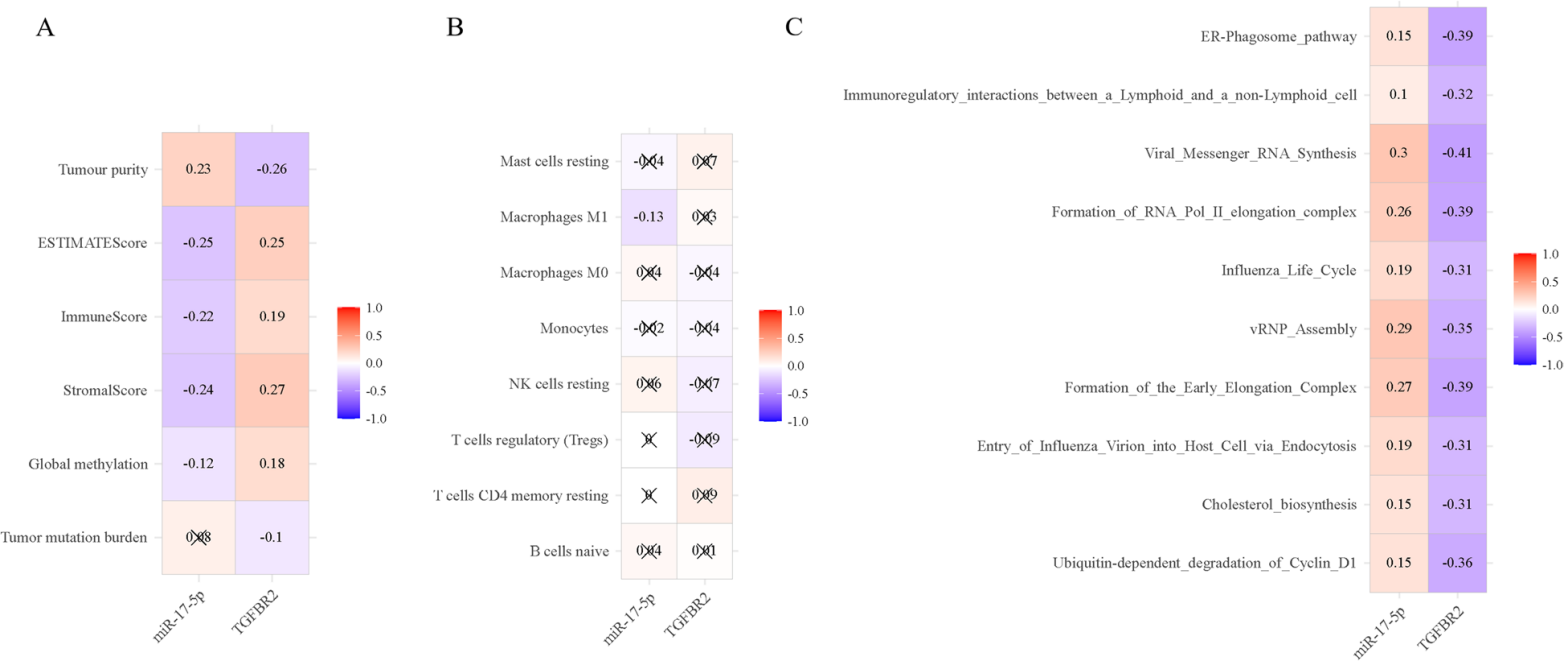
Pearson’s correlation analysis of immune-related phenotypes and regulatory pairs in TCGA-BLCA. **A**: ssGSEA; **B**: Immune cells; **C**: Global methylation, tumor mutation burden and tumor microenvironment factors

We downloaded the ssGSEA enrichment score of the TCGA-BLCA data from UCSC Xena and analyzed the correlation between the expression value of the miRNA-mRNA regulation pairs and the enrichment score. The results showed that the miRNA-mRNA regulation pairs correlated with the transport of Immunoregulatory interactions between a Lymphoid and a non-Lymphoid cell (Fig. 6A). We further analyzed its correlation with immune cells to explore its role in tumor immunity. We conducted a differential analysis of the immune cell data in TCGA-BLCA and found that 8 types of immune cells differed between tumor and normal tissues listed in Table S5. As shown in Fig. 6B, miR-17-5p correlated with macrophages M1. We used CIBERSORT to calculate the proportion of various immune cells in each TCGA-BLCA sample, ESTIMATE to estimate the proportions of stromal and immune components in tumor tissues, and obtain the methylation levels of CpG sites in TCGA-BLCA specimens from the UCSC Xena platform. As shown in Fig. 6C, the regulation pair of miR-17-5p and TGFBR2 has a specific correlation with TMB and tumor microenvironment but has nothing to do with DNA methylation.

## Discussion

In recent years, more and more studies have focused on the role of lncRNA, circRNA, mRNA, miRNA-mRNA, and epigenetic modifications in eukaryotes in the early detection, treatment, and prevention of cancer [22–26]. This study aimed to identify the regulatory pairs of miRNA-mRNA that play an essential role in the genesis and development of BLCA. We selected two miRNA and six mRNA datasets in the GEO database, all of which had the expression profile of miRNA or mRNA in BLCA and normal tissues. The GEO2R tool was used to screen DE-miRNAs and DE-mRNAs in the GEO database. The miRNAs with differences in 2 miRNA datasets were selected as DE-miRNAs, and the mRNAs with differences in 6 mRNA datasets were selected as DE-mRNAs. Expression profiles in cancer and normal tissues in the TCGA database were analyzed using “R-limma” and “R-edgeR” tools to screen for DE-miRNAs and DE-mRNAs. Ultimately, a total of 13 miRNAs (5 downregulated and 8 upregulated in BLCA tissues) and 181 mRNAs (72 upregulated and 109 downregulated in BLCA tissues) were selected as DE-miRNAs and DE-mRNAs. Seven miRNA-mRNA regulatory pairs were screened out after experimentally verifying miRNA-mRNA regulatory pairs from miRTarBase and Tarbase databases and Pearson’s correlation analysis of TCGA-BLCA. We further validated the expression level of 7 miRNA-mRNA regulation pairs in 28 pairs of FFPE BLCA tissues by qRT-PCR, and the pair of miR-17-5p and TGFBR2 were verified in the experiment.

In this study, miR-17-5p was upregulated in BLCA tissues confirmed by the previous studies [27]. miR-17-5p is involved in a wide range of biological processes. miR-17-5p is highly expressed in embryonic cells and its absence in mouse models results in hypoplasia [28, 29]. miR-17-5p promotes tumor proliferation by targeting PTEN and P21 [30]. It has been reported that miR-17-5p inhibits cell growth and promotes apoptosis of cervical cancer cells by targeting TP53INP1 [31]. In addition, miR-17-5p represses migration and invasion by directly targeting KCa1.1 and ERBB3 [32, 33]. miR-17-5p is a metastasis suppressor, and miR-17-5p plays an inhibitory role by targeting ETV1 and AIB1 [34, 35]. In this study, TGFBR2 was demonstrated to be downregulated in bladder cancer tissues. TGFBR2 has been reported to regulate the Hedgehog pathway and cervical cancer cell proliferation and migration by mediating SMAD4 [36]. Downregulation of TGFBR2 promotes the migration and invasion of CRC cells in colorectal cancer [37]. In conclusion, miR-17-5p and TGFBR2 play an essential role in tumor genesis and development.

This study also indicated that the miRNA-mRNA regulation pair had good diagnostic efficacy. We combined miR-17-5p and TGFBR2 as a panel using the logistic regression analysis and the AUC of the panel was 0.929 (95% CI: 0.885–0.972, *p* < 0.0001) in TCGA-BLCA and 0.767 (95% CI: 0.643–0.891, *p* = 0.001) in the external validation. The DCA is a method to evaluate prediction models and diagnostic tests that also showed that the miRNA-mRNA regulation pairs have an excellent diagnostic performance in distinguishing BLCA from normal patients. The expression of miR-17-5p and TGFBR2 showed no significant difference in age, gender, and TNM stage. The biomarker showed stable diagnostic performance in identifying BLCA patients regardless of the clinicopathological parameters.

Numerous studies support the critical role of immune infiltration in cancer development. The extent of T cell infiltration in tumors can predict a patient’s response to cancer immunotherapy [38]. M1 macrophages tend to polarize into M2 macrophages at advanced stages of the tumor and thus have cancer-promoting functions [39]. Correlation analysis between the miRNA-mRNA regulation pair and ssGSEA showed that the miRNA-mRNA regulation pair were related to Immunoregulatory interactions between a Lymphoid and a non-Lymphoid cell. Correlation analysis showed that miR-17-5p was negatively correlated with Macrophages M1. The tumor microenvironment plays a crucial role in the occurrence and development of tumors, and immune infiltration is one of the most essential features [40]. MiR-17-5p by targeting TGFBR2 could have an impact on the tumor microenvironment. Therefore, miR-17-5p and TGFBR2 have a necessary relationship with tumor immunity.

Although we carried out a comprehensive analysis and experimental verification of the miRNA-mRNA regulatory pairs involved in BLCA, there are still some deficiencies in this study, such as insufficient sample size and lack of studies on the mechanisms of DE-miRNAs and DE-mRNAs. Therefore, further studies on larger clinical samples and corresponding experiments are needed.

## Conclusion

In summary, we have identified a miRNA-mRNA regulatory pair (miR-17-5p and TGFBR2) that may be involved in the pathogenesis of BLCA and played an important role in disease diagnosis, tumor immunity, and other clinical applications.

## Supplementary Information

**Additional file 1: Figure S1.** GO and KEGG pathway analysis show the associated function of DE-miRNAs and DE-mRNAs. B: The KEGG and GO enrichment analysis of DE-miRNAs; C: The KEGG and GO enrichment analysis of DE-mRNAs.

**Additional file 2: Figure S2.** The expression of miR-17-5p and TGFBR2 in subgroups based on clinical pathological features and survival analysis of BLCA patients in TCGA. (Data are presented as mean±SEM; **p < 0.01). A: subgroups based on clinical pathological features; B: survival analysis.

**Additional file 3: Table S1.** The sequences of primers for candidate miRNAs and targeted mRNAs.

**Additional file 4: Table S2.** The list of DE-miRNAs and DE-mRNAs (up-regulated or down-regulated in BLCA).

**Additional file 5: Table S3.** Pearson's correlation analysis of miRNA-mRNA networks in TCGA-BLCA.

**Additional file 6: Table S4.** The sub-group analysis of mRNA or miRNA expression and muscle invasion or lymph node metastasis.

**Additional file 7: Table S5.** Immune cells differentiated between tumor tissue and normal tissue in TCGA-BLCA.

## Data Availability

The data that support the findings of this study are available from the corresponding author upon reasonable request.

## Abbreviations

miRNA: MicroRNA
BLCA: Bladder cancer
NMIBC: Non-muscle invasive bladder cancer
MIBC: Muscle-invasive bladder cancer
GEO: Gene Expression Omnibus
TCGA: The Cancer Genome Atlas
qRT-PCR: Quantitative reverse transcription-polymerase chain reaction
ROC: Receiver operating characteristic curve
DCA: Decision curve analysis
DE-miRNAs: Differential miRNAs
DE-mRNAs: Differential mRNAs
dbDEMC: database of Differentially Expressed MiRNAs in human Cancers
GO: Gene Ontology
KEGG: Kyoto Encyclopedia of Genes Genomes
ssGSEA: Single sample gene set enrichment analysis
ESTIMATE: Estimation of STromal and Immune cells in MAlignant Tumour tissues using Expression data
TMB: Tumor mutation burden
AUC: Area under curve
CI: Confidence interval
