# Supplementary figures and images for: Global analysis of miRNA-mRNA regulation pair in bladder cancer
Source: World J Surg Oncol. 2022 Mar 3;20:66. doi: 10.1186/s12957-022-02538-w (PMC8896384; doi:10.1186/s12957-022-02538-w)

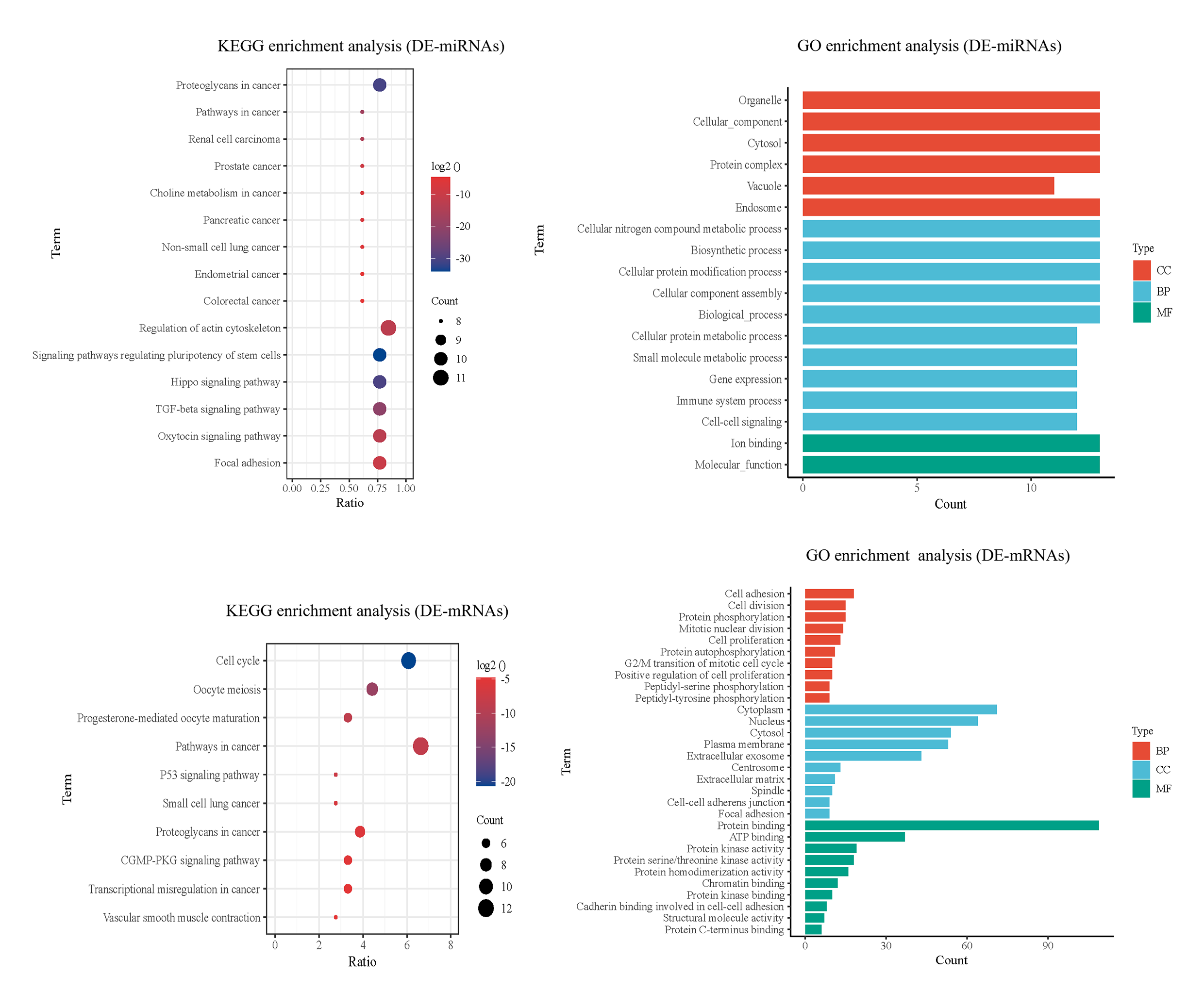

Supplement: Supplementary file 1 — Additional file 1: Figure S1. GO and KEGG pathway analysis show the associated function of DE-miRNAs and DE-mRNAs. B: The KEGG and GO enrichment analysis of DE-miRNAs; C: The KEGG and GO enrichment analysis of DE-mRNAs. [file 12957_2022_2538_MOESM1_ESM.tif]

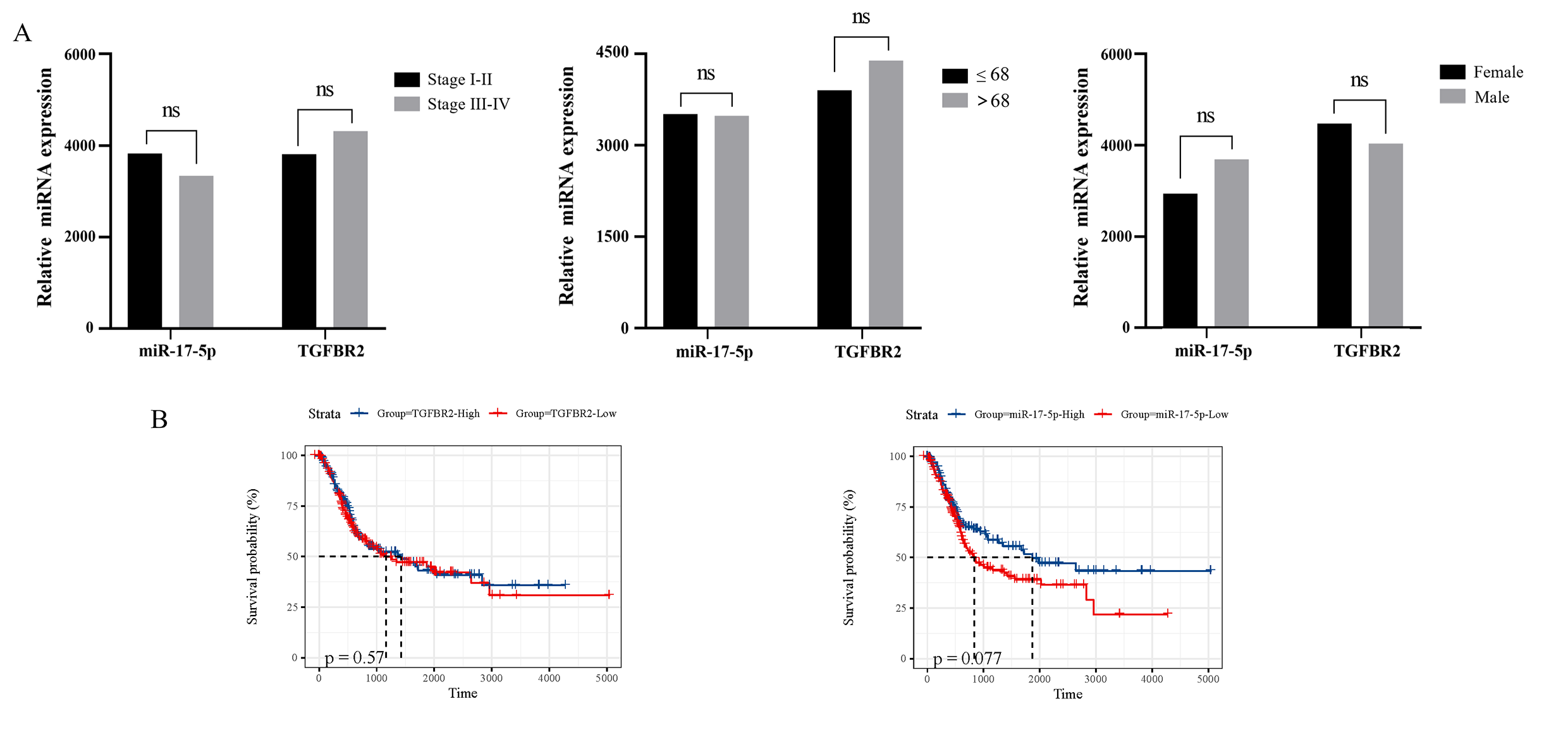

Supplement: Supplementary file 2 — Additional file 2: Figure S2. The expression of miR-17-5p and TGFBR2 in subgroups based on clinical pathological features and survival analysis of BLCA patients in TCGA. (Data are presented as mean±SEM; **p < 0.01). A: subgroups based on clinical pathological features; B: survival analysis. [file 12957_2022_2538_MOESM2_ESM.tif]
