# Supplementary material for: Global analysis of miRNA-mRNA regulation pair in bladder cancer
Source: World J Surg Oncol. 2022 Mar 3;20:66. doi: 10.1186/s12957-022-02538-w (PMC8896384; doi:10.1186/s12957-022-02538-w)
Supplement: Supplementary file 3 — Additional file 3: Table S1. The sequences of primers for candidate miRNAs and targeted mRNAs. [file 12957_2022_2538_MOESM3_ESM.docx]

| **Table S2: The list of DE-miRNAs and DE-mRNAs (up-regulated or down-regulated in BLCA)** | | | |
| --- | --- | --- | --- |
| **DE-miRNA (down)** | **DE-mRNA(up)** | **DE-miRNA(up)** | **DE-mRNA(down)** |
| miR-100-5p | ASPM | miR-141-3p | ABI3BP |
| miR-143-3p | AURKA | miR-210-3p | ACTN1 |
| miR-145-5p | AURKB | miR-93-5p | ADH1B |
| miR-195-5p | BUB1 | miR-182-3p | ANGPTL2 |
| miR-99a-5p | BUB1B | miR-130b-3p | ATP1A2 |
|  | CAMK2N1 | miR-17-5p | AXL |
|  | CBLC | miR-182-5p | BNC2 |
|  | CCNB1 | miR-934 | BOC |
|  | CCNB2 |  | C1ORF21 |
|  | CDC20 |  | C1S |
|  | CDC7 |  | C7 |
|  | CDK1 |  | CALD1 |
|  | CDK18 |  | CASQ2 |
|  | CENPF |  | CELF2 |
|  | CEP55 |  | CNTN1 |
|  | CHEK1 |  | COL14A1 |
|  | CKS2 |  | COLEC12 |
|  | DHCR7 |  | COPZ2 |
|  | DLGAP5 |  | CPXM2 |
|  | DTL |  | CRTAP |
|  | DUS1L |  | CYP2U1 |
|  | E2F3 |  | CYYR1 |
|  | EIF2AK1 |  | DCHS1 |
|  | EVPL |  | DDR2 |
|  | EZH2 |  | DENND5A |
|  | FABP6 |  | DENND5B |
|  | FASN |  | DIXDC1 |
|  | HIC2 |  | DPT |
|  | HMGB3 |  | EPHA3 |
|  | IGFBP3 |  | ERC1 |
|  | INPP4B |  | FAM50B |
|  | JUP |  | FAXDC2 |
|  | KIAA0101 |  | FBLN5 |
|  | KIF11 |  | FBXL7 |
|  | KRT7 |  | FERMT2 |
|  | KRT8 |  | FGL2 |
|  | LSR |  | FHL1 |
|  | MAPK13 |  | FNBP1 |
|  | MARCKSL1 |  | FZD7 |
|  | MCM7 |  | GPX3 |
|  | MELK |  | HSPB6 |
|  | MKI67 |  | ITM2A |
|  | NARF |  | ITPR1 |
|  | NCAPG |  | KCNJ8 |
|  | NOTCH3 |  | KCNMB1 |
|  | NUSAP1 |  | KCTD12 |
|  | PBK |  | LAMA2 |
|  | PLK4 |  | LAMB2 |
|  | plxna1 |  | LMO3 |
|  | PLXNB1 |  | LRCH2 |
|  | PMM2 |  | LRRK2 |
|  | PRC1 |  | LYVE1 |
|  | prkd2 |  | MAMDC2 |
|  | PRR11 |  | MAOB |
|  | PTK6 |  | MAP1A |
|  | RAB25 |  | MEF2C |
|  | SCAP |  | MEIS1 |
|  | SEMA3F |  | MFAP4 |
|  | SIRT7 |  | MSRB3 |
|  | SMC4 |  | MYOM1 |
|  | SOX4 |  | NAV3 |
|  | SPINT1 |  | NCAM1 |
|  | ST14 |  | NFIA |
|  | STAP2 |  | NFIX |
|  | STXBP2 |  | NR3C2 |
|  | TOP2A |  | NRP2 |
|  | TPX2 |  | OAT |
|  | TRAF4 |  | PAMR1 |
|  | TRIM24 |  | PDGFRA |
|  | TSTA3 |  | PDK4 |
|  | TTK |  | PDLIM3 |
|  | TYMS |  | PDZRN3 |
|  |  |  | PKDCC |
|  |  |  | PPP1R12B |
|  |  |  | PRKCB |
|  |  |  | PRRT2 |
|  |  |  | PRUNE2 |
|  |  |  | PTCH1 |
|  |  |  | PTRF |
|  |  |  | RGL1 |
|  |  |  | RGS1 |
|  |  |  | RGS2 |
|  |  |  | RHOJ |
|  |  |  | ROR1 |
|  |  |  | RPS6KA2 |
|  |  |  | RRN3P1 |
|  |  |  | SBSPON |
|  |  |  | SDPR |
|  |  |  | SGCA |
|  |  |  | SLC16A4 |
|  |  |  | SLC9A9 |
|  |  |  | SOBP |
|  |  |  | SORBS1 |
|  |  |  | SORBS2 |
|  |  |  | SPARCL1 |
|  |  |  | SPEG |
|  |  |  | SPON1 |
|  |  |  | SRPX |
|  |  |  | SSPN |
|  |  |  | STXBP6 |
|  |  |  | SYNM |
|  |  |  | SYNPO2 |
|  |  |  | SYT11 |
|  |  |  | TGFBR2 |
|  |  |  | THRA |
|  |  |  | WLS |
|  |  |  | ZCCHC24 |
|  |  |  | ZFHX4 |
|  |  |  | ZNF25 |
