# Supplementary material for: Global analysis of miRNA-mRNA regulation pair in bladder cancer
Source: World J Surg Oncol. 2022 Mar 3;20:66. doi: 10.1186/s12957-022-02538-w (PMC8896384; doi:10.1186/s12957-022-02538-w)
Supplement: Supplementary file 5 — Additional file 5: Table S3. Pearson's correlation analysis of miRNA-mRNA networks in TCGA-BLCA. [file 12957_2022_2538_MOESM5_ESM.docx]

| **Table S3： Pearson's correlation analysis of miRNA-mRNA networks in TCGA-BLCA.** | | | |
| --- | --- | --- | --- |
| ***miRNA (up)*** | ***mRNA (down)*** | ***p-value*** | ***r-value*** |
| miR-210-3p | NCAM1 | 0.1666 | -0.0678 |
| miR-93-5p | DENND5B | 0.6970 | 0.0191 |
| miR-93-5p | PPP1R12B | **0.0027** | **-0.1463** |
| miR-93-5p | TGFBR2 | **0.0000** | **-0.2089** |
| miR-130b-3p | PRUNE2 | **0.0077** | **-0.1301** |
| miR-130b-3p | TGFBR2 | **0.0046** | **-0.1383** |
| miR-17-5p | DENND5B | 0.3518 | 0.0457 |
| miR-17-5p | PPP1R12B | **0.0009** | **-0.1619** |
| miR-17-5p | TGFBR2 | **0.0000** | **-0.1998** |
| ***miRNA (down)*** | ***mRNA (up)*** | ***p-value*** | ***r-value*** |
| miR-195-5p | CDK1 | **0.0000** | **-0.2522** |
| miR-195-5p | E2F3 | 0.1032 | -0.0798 |
