# Supplementary material for: Global analysis of miRNA-mRNA regulation pair in bladder cancer
Source: World J Surg Oncol. 2022 Mar 3;20:66. doi: 10.1186/s12957-022-02538-w (PMC8896384; doi:10.1186/s12957-022-02538-w)
Supplement: Supplementary file 6 — Additional file 6: Table S4. The sub-group analysis of mRNA or miRNA expression and muscle invasion or lymph node metastasis. [file 12957_2022_2538_MOESM6_ESM.docx]

| **Table S4: The sub-group analysis of mRNA or miRNA expression and muscle invasion or lymph node metastasis.** | | | | | | | | | |
| --- | --- | --- | --- | --- | --- | --- | --- | --- | --- |
|  | **Variables** | **miR-195-5p** | **miR-93-5p** | **miR-130b-3p** | **miR-17-5p** | **CDK1** | **TGFBR2** | **PPP1R12B** | **PRUNE2** |
| **Mann-Whitney U** | Muscle invasion | 71.000 | 64.000 | 65.000 | 79.000 | 76.000 | 77.500 | 55.500 | 80.000 |
| ***p-value*** |  | 0.476 | 0.290 | 0.313 | 0.749 | 0.640 | 0.694 | 0.140 | 0.787 |
| **Mann-Whitney U** | Lymph node metastasis | 63.000 | 70.000 | 64.000 | 61.000 | 55.000 | 60.500 | 40.500 | 69.000 |
| ***p-value*** |  | 0.577 | 0.853 | 0.614 | 0.507 | 0.326 | 0.490 | 0.080 | 0.811 |
|  |  |  |  |  |  |  |  |  |  |
