# Supplementary material for: Global analysis of miRNA-mRNA regulation pair in bladder cancer
Source: World J Surg Oncol. 2022 Mar 3;20:66. doi: 10.1186/s12957-022-02538-w (PMC8896384; doi:10.1186/s12957-022-02538-w)
Supplement: Supplementary file 7 — Additional file 7: Table S5. Immune cells differentiated between tumor tissue and normal tissue in TCGA-BLCA. [file 12957_2022_2538_MOESM7_ESM.docx]

| **Table S5: Immune cells differentiated between tumor tissue and normal tissue in TCGA-BLCA.** | | | | |
| --- | --- | --- | --- | --- |
| **Cell type** | **Tumor** | **Normal** | **logFC** | ***p-value*** |
| Macrophages M0 | 0.0097 | 0.1272 | -3.7053 | 0.0000 |
| Mast cells resting | 0.1185 | 0.0438 | 1.4369 | 0.0003 |
| T cells CD4 memory resting | 0.1936 | 0.1225 | 0.6609 | 0.0016 |
| B cells naive | 0.0755 | 0.0343 | 1.1361 | 0.0019 |
| NK cells resting | 0.0035 | 0.0172 | -2.2843 | 0.0040 |
| Macrophages M1 | 0.0274 | 0.0633 | -1.2057 | 0.0057 |
| T cells regulatory (Tregs) | 0.0288 | 0.0483 | -0.7443 | 0.0304 |
| Monocytes | 0.0206 | 0.0153 | 0.4320 | 0.0316 |
